# The BioCASe Monitor Service - A tool for monitoring progress and quality of data provision through distributed data networks

**DOI:** 10.3897/BDJ.1.e968

**Published:** 2013-09-16

**Authors:** Falko Glöckler, Jana Hoffmann, Franck Theeten

**Affiliations:** † Museum für Naturkunde, Leibniz Institute for Research on Evolution and Biodiversity, Berlin, Germany; ‡ Royal Museum for Central Africa, Tervuren, Belgium

**Keywords:** BioCASe Provider Software, biodiversity informatics, OpenUp!, Global Biodiversity Information Facility (GBIF), Mapping Checker, Access to Biological Collection Data (ABCD) schema

## Abstract

The BioCASe Monitor Service (BMS) is a web-based tool for coordinators of distributed data networks that provide information to web-portals and data aggregators via the BioCASe Provider Software. Building on common standards and protocols, it has three main purposes: (1) monitoring provider’s progress in data provision, (2) facilitating checks of data mappings with a focus on the structure, plausibility and completeness, and (3) verifying compliance of provided data for transformation into other target schemas.

Herein two use cases, GBIF-D and OpenUp!, are presented in which the BMS is being applied for monitoring the progress in data provision and performing quality checks on the ABCD (Access to Biological Collection Data) schema mapping.

However, the BMS can potentially be used with any conceptual data schema and protocols for querying web services. Through flexible configuration options it is highly adaptable to specific requirements and needs. Thus, the BMS can be easily implemented into coordination workflows and reporting duties within other distributed data network projects.

## Introduction

In international biodiversity data initiatives a common goal is to build up distributed network infrastructures, e.g. in the *Global Biodiversity Information Facility* (GBIF; http://www.gbif.org) and the project *Opening up the Natural History Heritage for Europeana* (OpenUp!; http://www.open-up.eu). These distributed networks consist of natural history institutions or local aggregators, which provide their data to global aggregation portals, web services or other data consuming and transforming software. The main goal of these networks is to bring together locally distributed information and make it publically available. These infrastructures are often implemented on project bases and thus financed by third party funds. The particular success of the initiatives is often measured by progress indicators (e.g. number of data records or multimedia objects in a time period), which have to be recorded on a regular basis. Thus, continuous progress monitoring is indispensable for the production of high quality status reports. However, the greater the number of involved partners, the greater the challenge for the project coordination to monitor progress in data provision of each provider throughout the consortium and over the entire lifetime of a project. Secondly, monitoring key indicators relevant for the project, such as the amount of published data records per provider, involves recurring and time-consuming queries to each individual data source. Thus, it is highly desirable to facilitate progress monitoring by reducing the number of individually performed requests to decrease the workload and increase the time efficiency.

There are agreed data (exchange) standards and imposed required mandatory concepts depending on the focus and context of the data providing network. The project coordination is then also responsible for the quality assurance of the data provided and is obligated to continuously check the compliance and consistency of the data sources.

Furthermore, distributed networks (especially Europeana; http://www.europeana.eu) may involve transformations of the data structure into another schema prior to publication, e.g. if an intermediate level for data enrichment is included as in the case of the *OpenUp!* project (Berendsohn and Güntsch 2012, http://www.open-up.eu). Scientific and technical managers of these networks may benefit from a tool enabling a semi-automated method of verification that the data is prepared for transformation into the output schema.

Generally, technical staff and end-users would benefit from a service that gives an overview of the data providers and indicates that the participating provider services are on-line.

This paper presents a service tool, the *BioCASe Monitor Service* (BMS), which has been developed to match the above mentioned objectives. No other currently available service combines or covers these objectives into a single and easy-to-use tool. The BMS allows monitoring of data provision for a multi-partner consortium and facilitates the quality control of data sources that are connected to the network. The service is primarily intended for project coordinators of distributed networks and scientific or technical staff assessing the data quality, structure and completeness of published data according to the schemas used for data publication in the web.

The BMS has been developed through a collaborative effort between two data projects (*GBIF-D* and *OpenUp!*) dealing with biodiversity collection and observational data. However, it has the flexibility to be used by other communities of data providers as well.

## BioCASe Monitor Service

### Study area description

**Technical Background**

**Data provision in distributed networks**

In distributed biodiversity data networks the individual providing institutions (providers) manage their data supply by installing a technical infrastructure (e.g. a middleware) on top of their own databases. This is done in order to allow one or several Services or aggregating web portals to access the data via a central interface. Data are directly retrieved from the database located at provider side without resorting to a centralized public architecture for storage. With this approach, providers keep control over their data provision and are flexible in assigning institution-specific data policies. The middleware creates an abstraction layer by mapping the original data model (software- or institution-specific) to a common domain-specific (exchange) schema, e.g. the ABCD schema (http://wiki.tdwg.org/ABCD/).

In this step the provider can define the information flow by filtering the fields of the source database that are relevant concepts for the target network. The abstraction layer can then be directly harvested by domain specific harvesting tools, such as the GBIF *Harvesting and Indexing Tool* (HIT; http://code.google.com/p/gbif-indexingtoolkit/). Re-harvesting of the data source is then possible in order to make available the changes in the data.

In some projects or initiatives this step is iterated, for example if a transformation performed on an intermediate level is necessary for data provision in the domain-specific format to a more general (not domain-specific) structure. This facilitates data indexing and aggregation for web-portals and services, which publish the data for the end-users.

**Figure 1.**
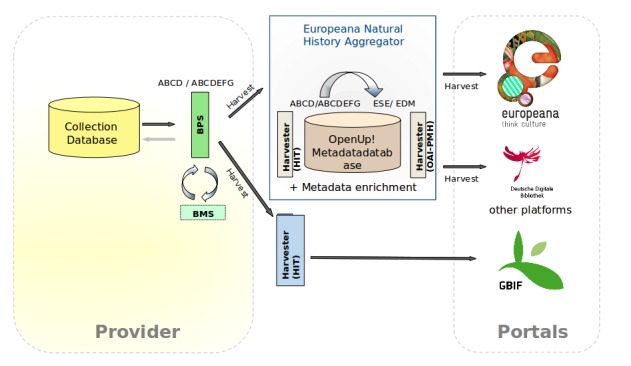
The architecture in distributed networks using the BioCASe Provider Software (BPS). This is illustrated just for one of many providers (left box) in the distributed network. The BioCASe Monitor Service (BMS) is used for checking the data compliance and requirements prior to the harvesting by the indexing tool (HIT). For the data provision to Europeana an additional transformation from the ABCD or ABCDEFG schema to the Europeana schema (ESE or EDM) is necessary. The requirements for the transformation are checked by the BMS at the provider side.

A complex data flow such as this is applied in the *OpenUp!* project (see Fig. 1), which harvests natural history collection data in ABCD(EFG) format (domain-specific) to pass the data from the so-called *OpenUp! Natural History Aggregator* along to the Europeana portal, which consumes ESE (Europeana Semantic Elements; http://pro.europeana.eu/ese-documentation) or EDM (Europeana Data Model; http://pro.europeana.eu/edm-documentation).

**BioCASe Provider Software**

The two biodiversity projects *GBIF-D* and *OpenUp!*, which are presented as examples in this paper (see section ‘Use cases’), use the *BioCASe Provider Software* (Biological Collection Access Service; Holetschek et al. 2012) as middleware. It supports the submission of collection and observational data to distributed networks. Furthermore, it enables the data provider to map their SQL capable databases to XML schemas and offers a *XML-over-HTTP* web-interface (see http://www.biocase.org/products/provider_software/, http://www.ibm.com/developerworks/xml/library/x-tiphttp/ and http://xmlrpc.scripting.com) for data access and data provision.

The *BioCASe Provider Software* (BPS) has been primarily developed for the data provision to its own European data portal *BioCASE* (http://search.biocase.org/europe). However, it is widely used within the biodiversity data community, because its compliance to the *Global Biodiversity Information Facility* (GBIF; http://www.gbif.org) was already assured during the development. It is natively configured for the ABCD schema (Access to Biological Collection Data; http://www.tdwg.org/standards/115/) and its derivatives (see below), as well as for DarwinCore (http://www.tdwg.org/standards/450/). However, the core component of the BPS is a generic wrapper library, which is capable of handling any conceptual XML schema. Therefore, there are no technical constraints to using the BPS in other areas of biodiversity informatics and natural science. A good example is the *Geoscientific Collection Access Service for Europe* (GeoCASE; http://www.geocase.eu/), which has been using the BPS since 2007 to aggregate data not only from paleontological, but also mineralogical and geological data sources.

Furthermore, the relatively small effort in setting up the BPS enables a wide range of possibilities for data exchange, because multiple software products are able to harvest and interpret the same data sources. In the example of GeoCASE, GBIF harvests the paleontological, but not the geological data.

**Domain-specific standards supported by the BioCASe Provider Software**

Official standard schemas and ontologies are designed and ratified by the scientific community i.e. the *Taxonomic Databases Working Group* (TDWG; http://www.tdwg.org/standards/). This assures that interoperability is warranted across different projects or initiatives.

The subsequent paragraphs briefly describe the data schemas which are mostly used in natural history context and are ordinarily supported by the BPS:

ABCD

The ABCD schema (*Access to Biological Collection Data*; currently in version 2.06) is a highly complex and extensible XML data model for information on natural history specimen collection and observational data (http://wiki.tdwg.org/ABCD/). It has a hierarchical structure and accommodates both atomized and free-text data. Thus, it can be used for a wide range of data in different qualities. ABCD 2.06 is an accepted schema of the *Biodiversity Information Standards TDWG* and can be used for standardized data exchange in biodiversity contexts, e.g. the data provision to GBIF. It is compatible with many existing data standards. A documentation of the particular elements can be seen at http://wiki.tdwg.org/twiki/bin/view/ABCD/AbcdConcepts.

- ABCD-EFG

The ABCD *Extension For Geosciences* (ABCD-EFG; http://www.geocase.eu/efg) was created to meet the specific needs of paleontological data. As there is a potential overlap in information on abiotic objects (e.g. stratigraphy, rock type) the extension was also designed to serve for geological and mineralogical collection data without any biological information. Consequently, the extension enables data provision of biological, paleontological and geological collection data at the same time. This reduces the efforts in mapping the data of natural history institutions, which curate physical objects of all three domains.

- ABCD-DNA

In order to provide DNA sample data together with their specimen data via the ABCD schema, generic concepts for supplementary contents (‘MeasurementsOrFacts’; see http://wiki.tdwg.org/twiki/bin/view/ABCD/AbcdConcept1339) would have to be implemented for DNA-specific values. As these values are relatively complex, the extension ABCD-DNA has been designed. It has been proposed to the TDWG as a new official standard schema for DNA data (http://www.tdwg.org/standards/640/).

DarwinCore

DarwinCore (often abbreviated DwC) is a set of elements from different ontologies and schemas (e.g. Dublin Core; http://dublincore.org/specifications/) for biodiversity and collection data. It has a flat structure that can be extended by domain-specific modules (e.g. geospatial, invasive species). DarwinCore is an accepted TDWG standard (http://rs.tdwg.org/dwc).

A stand-alone format, the DarwinCore Archive, is a self-described DarwinCore file. It is intended to ease the cataloguing of big datasets by processing them without requiring a live connection to the provider. This format is also useful for publications, because it can create a citable snapshot of a dataset. The BPS is able to convert ABCD data into the DarwinCore Archive format.

**The BioCASe Protocol**

The BPS communicates via its native query protocol (BioCASe Protocol; currently in version 1.3; http://www.biocase.org/products/protocols/). The BioCASe Protocol is an *XML-over-HTTP* format defined for sending SQL-like requests to the web service and receiving the respective response in XML format. It enables various possibilities to request metadata e.g. the elements provided (“capabilities” request), number of records (“search” request), number of unique values in a certain element (“scan” request), etc. These are relevant information for the requesting software, for example the *Harvesting and Indexing Tool* (HIT) used and developed by GBIF. The BioCASe Protocol queries the provided content for indexing and harvesting. Furthermore, it allows for partitioning of transmitted data by filtering and thus limiting the response. This is especially advantageous for handling large data sources, as well as for sequential harvesting. The BioCASe Protocol defines operators that allows diverse combinations of criteria to filter data sources (e.g. comparisons: ‘*Equals*’, ‘*isNull*’, ‘*lessThanOrEquals*’, ‘*greaterThan*’, etc.; negation: ‘*Not*’, ‘*isNotNull*’; combination: ‘*And*’, ‘*Or*’).

In addition, the protocol can combine the data with information on the operating system and the executed database queries, as well as warnings and error messages. Thus, it reports about the communication with the database and the status to give a comprehensible feedback on what is done in the background. This is particularly beneficial in the case where a malfunction needs to be debugged.

### Design description

**BioCASe Monitor Service**

The BioCASe Monitor Service (BMS) is a web-based tool programmed in PHP (*PHP: Hypertext Preprocessor*) and JavaScript. It uses the BioCASe protocol for automated compilations and output of information necessary for monitoring several BioCASe data sources simultaneously and performing quality checks of individual data sources.

The BMS consists of two interfaces: 1) the *BioCASe Monitor*, which is a catalogue of all registered data sources including some general metadata and relevant links, and 2) the *BioCASe Mapping Checker*, which gives a comprehensive overview of the respective mapping of a single data source and thus allows simple quality checks.

**The BioCASe Monitor**

**Figure 2.**
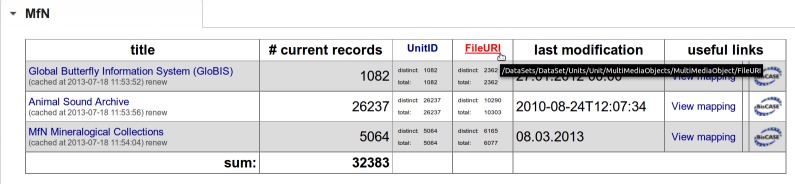
Each concept can be flexibly displayed with the total and distinct count of values.

The BioCASe Monitor is the entry point interface of the BMS. It consists of informative tables for each registered provider. These tables contain, at the minimum, a list of data sources, their access points (URI of the particular BPS data source), total number of records and date of last modification. For any concept of the provided data schema, a column can be displayed with the count of total and distinct values (see Fig. 2). These are flexible tables that can be customized in the configuration file depending on required progress indicators for monitoring. For example, the BioCASe Monitor can be used for rapid assessment of counts of taxon names, multimedia objects and locality names. Furthermore, it allows the detection of erroneous duplication of a unique identifier field.

**Figure 3.**
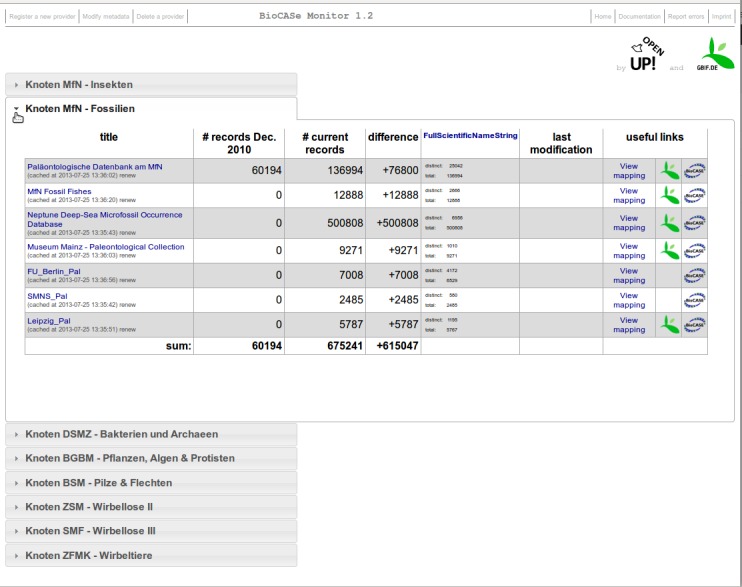
Collapsible or expandable boxes for grouping several data source entries (e.g. of the same provider) in the BioCASe Monitor.

The greater the number of data sources the greater is the necessity to group them into logical units. Therefore, the BioCASe Monitor offers the possibility of creating blocks of data sources, which can be configured as collapsible (and respectively expandable) boxes (see Fig. 3). As a result, the administrator is able to configure several independent groups of data sources with specific settings.

**Figure 4.**
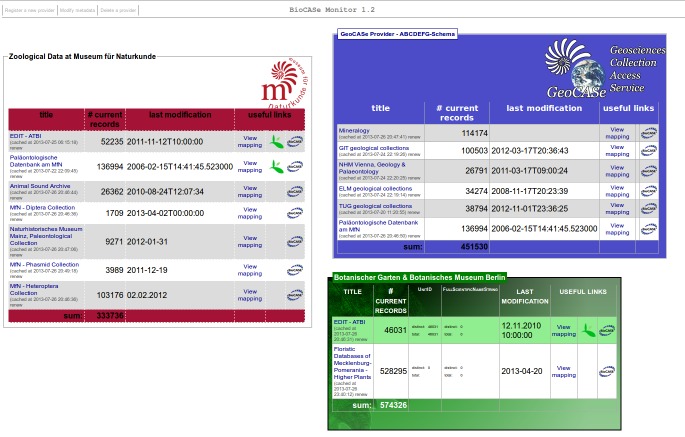
Examples for flexible CSS layouts of the BioCASe Monitor interface.

In addition, a customizable layout enables a free design for each block or table via *Cascading Style Sheets* (CSS; http://www.w3.org/Style/CSS/). This can be used to highlight boxes differently, and also provides the opportunity to adjust the layout to the project’s or institution’s corporate design (see Fig. 4). However, for these more advanced layout modifications, a basic knowledge of HTML-coding and CSS is needed.

Loading the BioCASe Monitor's provider overview can be time consuming depending on the number of data sources and requested concepts, but also due to long response time of the addressed servers. Eventually, this can result in a server timeout. In order to avoid this, the BioCASe Monitor sends requests asynchronously via AJAX (*Asynchronous JavaScript and XML*; http://www.w3schools.com/ajax/ajax_intro.asp) and successively adds this information to the overview. In addition, the response is stored in a caching system to improve the overall performance. Thus, reloading the overview page will only send requests to new sources or sources that have not been recently indexed. This approach avoids network congestion with redundant queries, in particular when datasets are not frequently updated and thus identical results are expected anyway. By default, the cache is renewed every 7 days after the last query to a provider. This interval can be changed in the configuration file. Refreshing the cache of individual data sources can also be triggered manually by clicking on a “renew” button.

In some cases (e.g. while dealing with big databases or focusing on a specific part of a dataset) it might be necessary to consider just a fraction of the data in a particular data source or to list subsets separately in the BioCASe Monitor overview. For this purpose, the BMS offers the possibility to pass filter criteria to the BioCASe Protocol, which allows fragmentation of a data source by defining different filters on the same access point. This feature increases the flexibility of coordinators to list desired metadata in a more structured way. Furthermore, it improves the performance when requesting large data sources. The link "View mapping" available for each data source refers to the second web interface of the BMS, the *BioCASe Mapping Checker.*

**BioCASe Mapping Checker**

**Figure 5.**
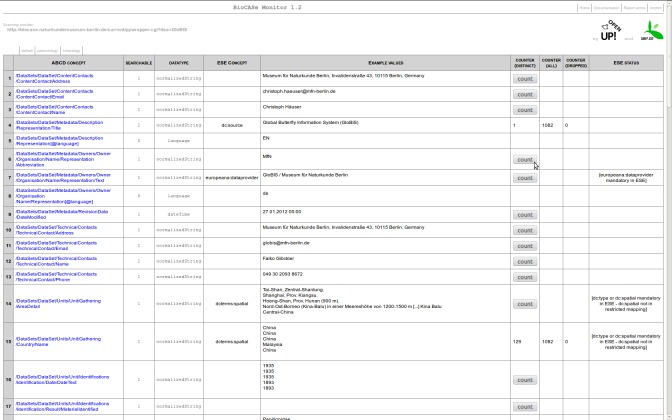
The BioCASe Mapping Checker with source concept x-path, searchability status, data type, target element for transformation check, example values, total and distinct count of values, count of dropped values and information on constraints of the target schema.

The Mapping Checker in the BioCASe Monitor Service lists the available XML elements of the mapped schema of a single data source. It also displays more details on each field e.g. the x-path, which identifies a particular concept, the expected data type of the schema and some sample value (Fig. 5). Furthermore, it enables the users to conduct plausibility checks by again offering the option to count the numbers of total and distinct values of any concepts (see also section “BioCASe Monitor”). An additional column contains the number of dropped values by the provider software. A dropped value, in the context of BioCASe, is a value present in the database but absent from the XML response. This is the case when a mandatory value is a) missing, b) dependent from another missing element at a higher hierarchy level, c) invalid (e.g. characters with wrong encoding), or d) outside a list of values defined in the XML schema. Thus, identifying the reason for dropped values is important for increasing the data quality.

The BPS can display values originating from a SQL database or from text directly inserted in the mapping. However, only values coming directly from an atomized database field can be filtered, which is flagged by the boolean attribute “searchable” in a data source’s capabilities. Thus, it is important to check the searchability of a given concept while debugging a mapping or identifying unexpected results of a request. The Mapping Checker facilitates this by simply requesting each element’s status and listing it in a column (see Fig. 5).

**Figure 6.**
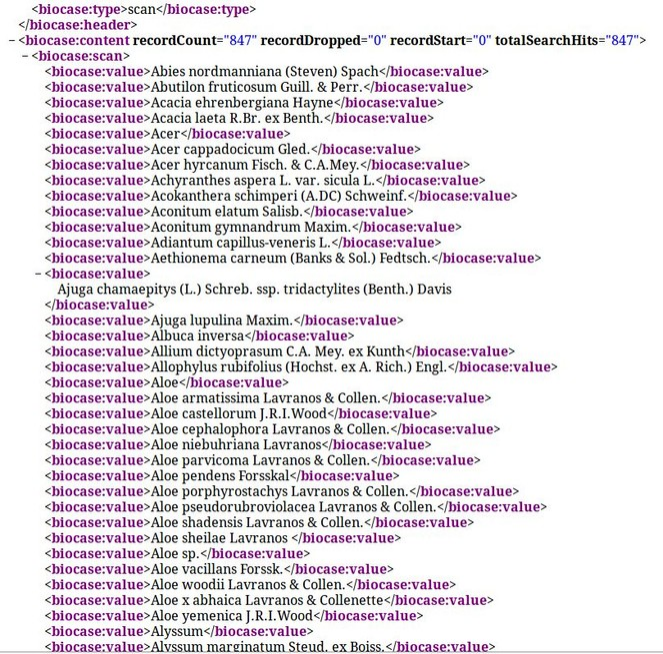
XML response of a scan request for taxon names in a BioCASe data source.

Plausibility checks of a mapping have to include the verification step that a XML response has the correct content. This is necessary, because an ABCD response can be technically correct, while the XML elements and attributes do not correspond to the schema definition regarding the contents. In the BioCASe Mapping Checker sample values of the first records of the XML response are displayed along with the corresponding concept. That way the user gets an impression of values of specific concepts in the data source. This feature is essential for quality control by the domain expert. A more detailed insight into the concept's values is provided by a hyperlink in the x-path column. By clicking this link, a scan request is performed in order to display all unique values of the respective concept (see Fig. 6). Thus, the direct XML output of a scan request on e.g. taxon names, localities, multimedia object URIs, etc. can be easily assessed.

Data aggregators can use different XML schemas, depending on the purpose and scope of the service consuming them. This often implies a transformation between the provider’s original XML schema and a target schema. An example is Europeana (see Fig. 1 and use case OpenUp!), which aggregates content from sources of different disciplines. These data sources do not necessarily share the same exchange formats. In this situation a crucial step in checking a data source’s plausibility is to test whether the mapping of the source schema meets the target schema’s requirements. In order to validate this, the BioCASe Mapping Checker checks the presence of the source elements according to the requirements of the target concepts. Consequently, the content provided is ready for a transformation made by XSL-T (or similar techniques; http://www.w3.org/TR/xslt). In the case that a concept of the source schema corresponds to several concepts in the target schema (and vice versa), the Mapping Checker is able to check whether an element is mandatory and/or required to be unique at the target side. According to the results of the mapping checks it highlights eventual issues, for example, missing target elements. Furthermore, it is possible to switch between different predefined sets of requirements or target schemas.

The options provided with the Mapping Checker focus on single data sources, because it is designed as a stand-alone service interface totally independent from the BioCASe Monitor. It can be used for any given BioCASe data source URI that is transferred via a parameter in the service’s endpoint URL (e.g. http://gbif.naturkundemuseum-berlin.de/biocaseMonitor/services/getConceptsInfo.php?url=http://biocase.naturkundemuseum-berlin.de/current/pywrapper.cgi?dsa=mfn_pal_fish). This makes the Mapping Checker a useful tool even for non-network partners and end-users, who may want to perform checks on their mapping individually. It is also helpful in the process of setting up new mappings of network providers, as this includes a large amount of testing.

The output of the BioCASe Mapping Checker is HTML by default, but XML output is supported alternatively. This makes the output suitable for further processing by other software (e.g. R-scripts for more elaborated statistics; Stowell 2012; http://www.r-project.org) and not only by humans.

**BioCASe Monitor Service – Use Cases**

**OpenUp!**

In the OpenUp! project, the BMS is used for the overall organization and monitoring of the progress (http://edit.africamuseum.be/biocasemonitor) in providing ABCD and ABCD-EFG formatted data through the BioCASe Provider Software to Europeana (http://www.europeana.eu). In OpenUp!, the BMS mostly addresses the needs of the coordinators of the two content providing work packages for botanical and zoological data. The BioCASe Monitor allows the coordinators to set up and modify a dynamic and commonly shared list of all data providers and data sources. There are 20 data providers hosting more than 90 data sources (as of July 2013) in the project.

In OpenUp!, the main focus of data provision is on making multimedia objects and associated metadata accessible through the Europeana platform. Therefore, the counts of total multimedia resource references, which are represented as URIs (and thus called *FileURI* s in the ABCD schema; see http://wiki.tdwg.org/twiki/bin/view/ABCD/AbcdConcept0774), is the most important information for reporting and represents a key performance indicator. However, there is a necessary distinction between ‘total’ FileURIs and ‘distinct’ FileURIs, because in some cases the same multimedia objects are associated with more than one data record in a data source. By simply counting the ‘total’ number of FileURIs these multimedia objects are counted multiple times and thus distort the numbers for reporting. The relation between distinct and total values for the FileURIs can be easily assessed in the BioCASe Monitor.

Furthermore, the BioCASe Monitor offers the possibility to export the compiled numbers and information in a tab-delimited format. This is especially helpful for the production of the regular reports in the project (for example Hoffmann et al. 2012, Hoffmann and Hirschfeld 2013).

Another main task in the OpenUp! project is the assurance of the quality standards defined in the project and imposed by the use of the ABCD standard, as well as the assurance of the compliance of the provided data with the target schema *Europeana Semantic Elements* (ESE; http://pro.europeana.eu/ese-documentation) and *Europeana Data Model* (EDM; http://pro.europeana.eu/edm-documentation). Therefore, the BioCASe Mapping Checker, which is directly linked in the BioCASe Monitor (see above), allows a simple but efficient overview of the mapping of each data source. Here, the coordinator can check the completeness of the mapping and correct usages of ABCD concepts in relation to the displayed sample values.

The completeness of the mapping in terms of mandatory concepts for the harvesting, but also the transformation procedure, is automatically checked for a default (zoology and botany), a mineralogy and a paleontology setting. This check is done against the transformation rules agreed upon in the project (Zagorsek et al. 2012). A message box appears below the mapping if, for example, mandatory concepts are missing, but required for a successful harvesting event or a valid transformation from ABCD-EFG to ESE / EDM.

The flexibility in the implementation of the BMS into existing workflows has been proven in the OpenUp! project by finding a solution for a particular issue: ABCD is centered on two concepts, the metadata of a single collection (represented in the element ‘Dataset’; http://wiki.tdwg.org/twiki/bin/view/ABCD/AbcdConcept0001) and its respective collection objects (represented as ‘Units’; http://wiki.tdwg.org/twiki/bin/view/ABCD/AbcdConcept0135), which are often identified by a unique inventory number. In the context of OpenUp!, if these two elements are linked together in the source database without proper normalization, the element ‘Dataset’ is repeated as many times as there are unit elements. This prevents the correct indexation of the provider by the harvesting service. Consequently, the BMS has been configured to check for this repetition and to provide a warning message, if the constraint is not respected.

The Mapping Checker allows not only the coordinator to perform the final validation before the official release for a test harvest by the *OpenUp! Natural History Aggregator.* It also has proven to be a helpful tool for the providers to perform initial checks of data source mappings during the setup phase. Furthermore, it facilitated communication about necessary changes between the coordinator and the individual provider or the technical staff of a provider and the scientist responsible for a particular the data source. Thus, the BioCASe Monitor Service is an essential component in the OpenUp! infrastructure.

After the end of the funding period of the project, providers will be able to use the BioCASe Mapping Checker as a tool facilitating the correct setup of their data sources according to the Europeana standards. In addition, the BioCASe Monitor will show the intended increase of objects provided to Europeana as well as the expansion of the provider network.

**GBIF-D**

In Germany data mobilization and provision to the international GBIF network is coordinated by the GBIF-D project (http://www.gbif.de), which itself is further subdivided into 8 thematic nodes and a coordination team. A node in GBIF-D represents a specific taxonomic focus, which is thought to increase noticeably the quality of the data. Nevertheless, coordinating these subnodes and creating a common overview of the data sources in the German network increases the organisational effort tremendously as opposed to a single node organisation. The BioCASe Monitor facilitates the aggregation of metadata for the project coordination team and automatically provides relevant information for reporting, such as a complete overview of all connected data sources in GBIF-D including the overall number of records (http://www.gbif.de/statistics). The schema used in GBIF-D is the ABCD schema and its extensions (see above). In order to communicate about the extensive amount of fields in the ABCD schema, it is easy for the nodes staff sending the BioCASe Mapping Checker link to the database managers and owners for demonstrations, discussions and improvements of the mapping of a respective database.

In the BioCASe Monitor instance of GBIF-D (http://www.gbif.de/statistics) each thematic node is represented by a collapsible block, which is equipped with an additional column to display the number of biodiversity data records that have been provided in the first funding period, before December 2010. In fact, the increase of the data records provided since 2010 is one of the evaluation criteria set by the national funding agency. Additionally, a simple but specific modification highlights this increase in data records by displaying the automatically calculated difference between the number of data records from the former and current funding period.

A column for useful links has been created to manually enrich rows per data source with additional hyperlinks. For each column only a variable in the configuration file needs to be filled with the desired URL in order to create a new hyperlink. Thus, the overview can be supplemented with any related references. For example, once the data is harvested by GBIF international, there is a reference available (e.g. http://data.gbif.org/datasets/resource/14719) allowing direct link to GBIF data that have undergone additional plausibility and quality checks, mainly on the geography and taxonomy. If some data records might be dropped or ignored during the harvesting process, e.g. if a taxon name does not match any entry in the taxonomic backbone, the number of records in the GBIF portal could differ from the one in the BioCASe Monitor. Thus, linking to the GBIF target reference is a helpful shortcut for GBIF-D as it facilitates subsequent plausibility and data quality checks by directly comparing the number of records, taxa, geo-references etc. of the source with the international portal reference.

As *GBIF-D* is focused on installing and extending the distributed infrastructure for biodiversity data provision (with the BioCASe Provider Software) in Germany, the number of data records will continue to grow even after the ongoing funding period. This ongoing increase will be reflected by the BioCASe Monitor in future as it will renew its cache continuously without additional maintenance.

**Discussion and Outlook**

The two described use cases demonstrate the wide ranging possibilities of the BioCASe Monitor Service for coordinators and providers in distributed data networks. The tool facilitates plausibility and compliance checks by providing flexible compilations of metadata and numbers. For regular reporting duties in distributed network projects, metadata can simply be exported or copied to be pasted into the respective document. Depending on the user's demands, more export formats could be added at a later stage (e.g. Microsoft Excel).

Due to the projects’ premises and contexts for which the BMS has been created, the focus is primarily on the usage with the ABCD schema and its extensions. However, the BioCASe Monitor Service is an easily adaptable tool and thus can be applied also for other schemas (e.g. DarwinCore) used in the area of (biodiversity) informatics. As the core part is the BioCASe Protocol, the same standard format is used for communication with the web services. Consequently, there are no direct limitations regarding different schemas as long as they are implemented in the BioCASe Provider Software. It is planned to undertake some tests to demonstrate the technical feasibility of this.

The configuration of the Monitor Service is organized in a plain text file by using the easily comprehensible INI format (Robichaux 1998). The elements counted by the BioCASe Monitor, the correspondences between the target and source schemas checked by the Mapping Checker, as well as the constraints (mandatory field, unique field) are defined in delimited text files having a simple and easy-to-modify structure. Thus, the tool’s great flexibility through setting different preferences can be exploited even by non-informaticians. Nevertheless, an already included but still relatively basic approach for an administration backend is planned to be further developed to dispense with editing the configuration files.

The feature of having a caching mechanism in the BioCASe Monitor is very effective for both, the reduction of superfluous traffic at the provider’s end point and the performance improvement of the Monitor Service. Simple text files are used for storing the cached values in order to avoid the requirement of having an additional database. These cache files, which are tagged by a timestamp, will be re-used for a planned module that will create graphics and diagrams of the progress of data provision automatically. This more sophisticated feature is expected to be a perfect addition to the service regarding the implementation in the regular project reports.

It can be concluded that the BioCASe Monitor Service fills a gab in the management of distributed (biodiversity) data networks as it facilitates many workflows concerning the coordination of the providers and monitoring their progress in data provision. It is open for adaptations and functionality extensions for both, the network members and the end-users of data portals.

### Funding

The OpenUp! project is a Best Practice Network, co-funded by the European Commission under the eContentplus programme, as part of the i2011 policy. GBIF-D is funded as a joint research project by the German Federal Ministry of Education and Research (BMBF).

## Web location (URIs)

Wiki: http://biocasemonitor.biodiv.naturkundemuseum-berlin.de/

Download page: http://biocasemonitor.biodiv.naturkundemuseum-berlin.de/index.php/Download

## Technical specification

Platform: platform independent web-server with PHP

Programming language: PHP, JavaScript, HTML, CSS

Interface language: English

## Repository

Type: SVN

Browse URI: http://svnrepo.biodiv.naturkundemuseum-berlin.de/repos/biocasemonitor/

## Usage rights

### Use license

Creative Commons CCZero
